# Pleiotropic Cholesterol Signaling in *Drosophila* and Mammalian Systems

**DOI:** 10.3390/metabo16040220

**Published:** 2026-03-27

**Authors:** Yueyang Kang, Muhammad Atif, Youngseok Lee

**Affiliations:** Department of Integrative Biotechnology, Kookmin University, Seoul 02707, Republic of Korea; makikyy@kookmin.ac.kr (Y.K.); itsatifcontact@koomin.ac.kr (M.A.)

**Keywords:** cholesterol, feeding behavior, nutrient sensing, sterol auxotrophy

## Abstract

Growth, reproduction, and survival are fundamental biological priorities that animals balance by evaluating dietary cues. Cholesterol occupies a unique position among nutrients, serving both as a structural component of cellular membranes and as a precursor for steroid hormones, yet its regulation differs fundamentally across taxa. In mammals, cholesterol availability is buffered by endogenous synthesis and post-ingestive metabolic control. In contrast, insects have evolutionarily lost sterol biosynthesis and are therefore sterol auxotrophs that rely entirely on dietary sources. Here, we synthesize current understanding of cholesterol biology in *Drosophila melanogaster*, with a focus on sterol auxotrophy, life-stage–specific allocation, and endocrine and lifespan outcomes in a comparative framework. We highlight cholesterol not only as a metabolic substrate but also as a sensory-encoded nutrient that shapes feeding behavior. We further examine how lipophorin (Lpp)-mediated transport, Niemann–Pick type C proteins, ATP-binding cassette transporters, and the nuclear receptor DHR96 coordinate systemic sterol distribution and endocrine output in the absence of endogenous synthesis. By integrating these mechanisms across development, we illustrate how cholesterol availability governs larval growth, ecdysteroid production, adult reproduction, neural function, and lifespan through coupled endocrine and nutrient-signaling networks. This review positions cholesterol as a multifunctional signal linking sensory perception, metabolic regulation, and life-history strategy in sterol-auxotrophic insects, offering a framework for understanding how evolutionary loss of biosynthetic capacity reshapes nutrient sensing and homeostatic control.

## 1. Introduction

**Figure 1:**
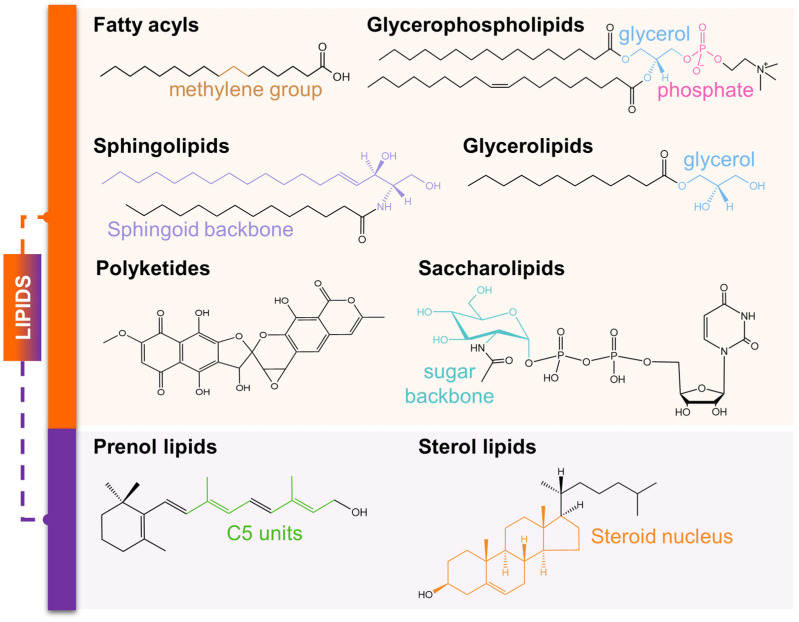
Lipid classification based on structure and biosynthesis. Lipids are categorized into eight major classes. Lipid classes derived from the condensation of ketoacyl subunits (orange block) include fatty acyls (hexadecanoic acid), glycerolipids (1-dodecanoyl-sn-glycerol), glycerophospholipids (1-hexadecanoyl-2-(9Z-octadecenoyl)-sn-glycero-3-phosphocholine), sphingolipids (N-(tetradecanoyl)-sphing-4-enine), saccharolipids (UDP-N-acetyl-αD-glucosamine), and polyketides (griseorhodin A). In contrast, sterol lipids (cholesterol) and prenol lipids (vitamin A) (purple block) originate from the condensation of five-carbon (C5) isoprene units through the mevalonate or methylerythritol phosphate pathways. Representative structures are shown for each class to highlight fundamental backbone architectures. The aforementioned grouping is based on LIPID MAPS classification framework.

To sustain growth, homeostasis, and reproduction, organisms require a coordinated supply of macro- and micronutrients [1]. Among these, lipids are pleiotropic regulators of structure, development, metabolism, and reproduction [2]. Following the LIPID MAPS classification [3,4] (Figure 1), cholesterol is the most abundant and biologically significant sterol in animals [5]. It serves a multifaceted role in metabolic and homeostatic regulation [6,7,8,9,10,11], development [12,13,14], physiology [15,16,17], aging [18,19,20,21], immunity [22,23,24], and cancer [25,26,27]. This wide influence reflects its dual nature: as an essential structural component modulating fluidity, permeability, and membrane-protein organization [28], and as a metabolic precursor [29,30] for bile acids and multiple classes of steroid hormones—including aldosterone, progesterone, cortisol, estrogen, and testosterone—that coordinates endocrine homeostasis [31,32]. These roles underscore the biochemical versatility of cholesterol and the evolutionary importance of maintaining its precise regulation across diverse organisms.

In mammals, cholesterol regulates cellular homeostasis as a membrane stabilizer lipid, a component of lipid rafts [33] and a precursor for steroid hormones and oxysterol signaling molecules [34,35,36]. Levels are maintained through an integrated *de novo* synthesis, lipoprotein-mediated uptake, intracellular transport, and biliary excretion [10,37]. Perturbations in this system underlie major diseases including atherosclerosis, metabolic syndrome, neurodegeneration, and certain cancer [23]. This complexity provides a comparative framework for understanding sterol regulation in other taxa.

Unlike mammals, arthropods including insects have evolutionarily lost key enzymes and are therefore sterol auxotrophs reliant on dietary sterols [38,39]. To combat this constraint, insects must acquire, convert, and transport exogenous sterols to fulfill the physiological and structural demands. Among dietary sterols, cholesterol is the essential precursor for ecdysteroid synthesis, requiring lipoprotein-mediated uptake and Niemann–Pick type C-dependent trafficking [40,41,42,43,44]. Beyond development, it modulates reproduction—affecting oogenesis [45], vitellogenesis [46], and juvenile hormone signaling [47]—influencing insulin-like peptide secretion, lifespan, and somatic fertility trade-offs [48,49]. Thus, cholesterol is a pivotal regulator orchestrating insect endocrine function and longevity.

*Drosophila melanogaster* remains pivotal to nutritional biology [50], elucidating pathways for sensing and regulating both macronutrients [51,52,53,54,55,56,57,58,59,60,61,62], and micronutrients [48,63,64,65,66,67,68,69,70,71,72,73,74]. Given its status as a sterol auxotroph, fruit flies must identify dietary sterols via gustatory receptor neurons (GRNs) acting as the primary interface [75], linking external sensory cues to the internal metabolic requirements for growth and reproduction. Consequently, cholesterol serves a dual role: a dynamic sensory ligand that drives behavioral choices [74] and a vital metabolic substrate sustaining physiological homeostasis [10].

This review synthesizes cholesterol biology with a focus on the interplay between sensory perception and metabolic utilization. By utilizing mammalian regulation as a comparative framework, we provide an overview of how *Drosophila* acquires and processes this vital lipid across developmental stages. Ultimately, this synthesis provides a blueprint for understanding the multi-dimensional nature of sterol biology and nutrient-sensing architectures.

## 2. Comparative Analysis of Cholesterol Gustation in Mammals and Insects

Animals sense their chemical environment using the chemosensory modalities of olfaction and gustation [76,77]. Taste systems enable animals to evaluate the chemical composition and nutritional value of ingested substances through receptor-defined sensory pathways. In mammals, taste detection is mediated by specialized taste receptor cells organized into taste buds [78]. Sweet and umami tastants are detected by TAS1R heterodimeric receptors [79,80,81], bitter compounds by members of the TAS2R receptor family [82,83], amiloride-sensitive salt by epithelial sodium channels (ENaC) [84], sour stimuli by PKD2L1-expressing cells [85], and carbonation by carbonic anhydrase IV–dependent mechanisms [86]. Together, these receptor-defined cell populations establish a modular sensory architecture optimized for the selective detection of chemically diverse tastants and for linking specific nutrient classes to distinct behavioral outputs.

**Figure 2:**
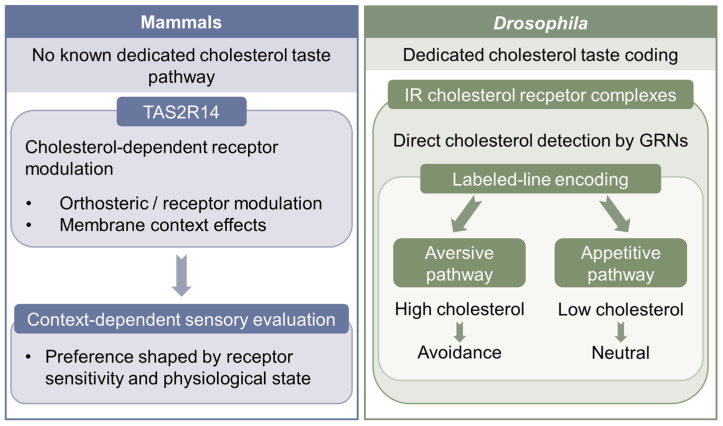
Distinct sensory logic of cholesterol evaluation in mammals and *Drosophila*. Mammals lack a dedicated cholesterol taste pathway, and cholesterol-related sensory effects are thought to arise mainly through receptor modulation and membrane-context effects that influence preference in a physiological-state-dependent manner. In *Drosophila*, cholesterol is detected directly by dedicated IR-based gustatory pathways in GRNs, producing labeled-line outputs in which high cholesterol is aversive, whereas low cholesterol is neutral. This figure summarizes the contrast between indirect modulatory sensing in mammals and direct gustatory coding in insects.

Within this canonical framework, cholesterol has traditionally been regarded as a post-ingestive metabolic signal rather than a primary sensory cue. However, accumulating evidence indicates that cholesterol can directly influence mammalian gustatory systems, primarily by modulating receptor function rather than through a dedicated “cholesterol taste” pathway [87,88,89]. Behavioral studies in mice demonstrate a rapid preference for cholesterol-enriched diets, consistent with the existence of taste-associated or early post-ingestive mechanisms capable of detecting dietary cholesterol. Using a dual-diet test paradigm, recent studies showed that wild-type mice preferentially consumed cholesterol-rich chow and that this preference was altered in ATP-binding cassette transporter A1 (ABCA1)-deficient animals, implicating physiological cholesterol handling in shaping cholesterol-driven feeding behavior rather than passive caloric reward [90]. At the molecular level, recent structural and functional studies identify bitter taste receptors—most notably TAS2R14—as direct targets of cholesterol. Cryo-electron microscopy structures reveal that cholesterol can occupy the canonical ligand-binding pocket of TAS2R14, acting as an orthosteric modulator that enhances receptor activation by bitter agonists [87,88]. Complementary functional analyses in human airway smooth muscle and epithelial cells demonstrate that membrane cholesterol regulates TAS2R14 signaling through defined cholesterol–receptor interaction motifs, with cholesterol depletion impairing and replenishment restoring receptor responsiveness [89]. Collectively, these findings support a model in which cholesterol does not constitute a classical tastant with an independent perceptual identity in mammals, but instead tunes gustatory receptor sensitivity in a context-dependent manner, thereby linking dietary lipid availability and membrane composition to sensory evaluation and downstream physiological state. Because much of this mechanistic work has been performed in airway and heterologous systems, an important open question is how strongly these cholesterol–TAS2R14 interactions contribute to oral taste perception in vivo. A comparative overview of cholesterol gustation strategies in mammals and *Drosophila* is provided in Figure 2.

These mammalian findings contrast sharply with insects, in which chemical detection has now been shown to rely on dedicated gustatory receptors rather than receptor modulation alone [91]. Direct evidence that insects can taste cholesterol has recently been provided in *Drosophila*. Using behavioral, genetic, and receptor-reconstitution approaches, flies are largely indifferent to low concentrations of cholesterol but robustly avoid higher levels, establishing cholesterol as a *bona fide* gustatory stimulus rather than a purely post-ingestive metabolic factor [74]. Cholesterol avoidance is mediated by a subset of GRNs that partially overlap with bitter-sensing neurons, indicating engagement of aversive taste circuitry. At the molecular level, cholesterol detection requires specific combinations of ionotropic receptor (IR) family members, with disruption of individual subunits abolishing cholesterol-evoked avoidance. Functional reconstitution experiments revealed two distinct multimeric cholesterol receptor complexes composed of three shared IR subunits and one receptor-specific subunit; strikingly, ectopic expression of these cholesterol receptors in sugar-responsive GRNs was sufficient to convert cholesterol from an aversive to an attractive stimulus, demonstrating that the behavioral valence of cholesterol is determined by GRN identity rather than by the chemical itself. Together, these findings establish IRs as a molecular basis for cholesterol gustation in insects and reveal a receptor-defined mechanism fundamentally distinct from cholesterol-sensitive modulation of bitter receptors observed in mammals [74].

This dedicated cholesterol taste pathway likely reflects the ecological pressure imposed by sterol auxotrophy, enabling *Drosophila* to avoid sterol-rich environments that could disrupt finely tuned sterol homeostasis [29,74]. Cholesterol taste encoding is compared across mammalian and insect taxa (Table 1). Taken together, mammalian and insect taste systems illustrate two distinct solutions to integrating cholesterol into chemosensory control of feeding [74,87,88,89]. In mammals, cholesterol primarily shapes gustatory signaling by modulating receptor function and membrane properties, consistent with its role as a systemic metabolic regulator [87,88,89]. In *Drosophila*, by contrast, cholesterol is encoded as a discrete gustatory stimulus by dedicated IR complexes whose activation recruits either aversive or appetitive lines, reflecting the stringent sterol economy of an auxotrophic organism [74].

## 3. Sterol Auxotrophy Underscores a Profound Regulatory Divergence Between Insects and Mammals in Cholesterol Metabolism

**Figure 3:**
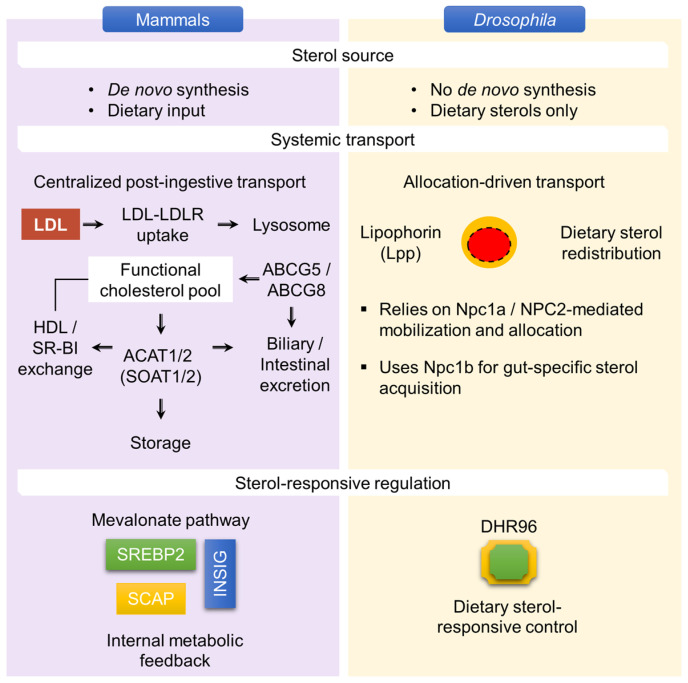
Sterol auxotrophy drives divergent cholesterol-regulatory strategies in mammals and *Drosophila*. Mammals regulate cholesterol through an internally buffered network involving *de novo* synthesis, dietary input, systemic transport, intracellular trafficking, and sterol-responsive control. In mammals, circulating low-density lipoprotein (LDL) is internalized through low-density lipoprotein receptor (LDLR)-mediated uptake and trafficked to lysosomes, thereby contributing to the functional cholesterol pool. Cholesterol can also be exchanged through high-density lipoprotein (HDL) and scavenger receptor class B member 1 (SR-BI), esterified by acyl-CoA: cholesterol acyltransferase 1/2 (ACAT1/2; also known as sterol O-Acyltransferase 1/2, SOAT1/2) for storage, or exported through ATP-binding cassette subfamily G member 5 and member 8 (ABCG5/ABCG8) for biliary or intestinal excretion. Sterol-responsive regulation is further coordinated by sterol regulatory element-binding protein 2 (SREBP2), SREBP cleavage-activating protein (SCAP), and insulin-induced gene proteins (INSIG) within the mevalonate pathway. In contrast, *D. melanogaster* lacks *de novo* cholesterol synthesis and depends entirely on dietary sterols. In insects, sterol homeostasis is therefore organized around external acquisition, lipophorin (Lpp)-mediated systemic delivery, Niemann–Pick type C1a (Npc1a)/Niemann–Pick type C2 (NPC2)-dependent intracellular mobilization, Niemann–Pick type C1b (Npc1b)-mediated gut sterol acquisition, and *Drosophila* hormone receptor 96 (DHR96)-dependent transcriptional regulation, which together connect environmental sterol supply to organismal physiological outputs.

In mammals, cholesterol homeostasis is maintained through multifaceted metabolic processes that operate primarily within a post-ingestive regulatory framework. Insects, by contrast, are sterol auxotrophs and therefore depend directly on dietary sterols. These fundamentally distinct cholesterol-regulatory architectures in mammals and *Drosophila* are illustrated in Figure 3.

Consistent with this divergence, recent studies in *Drosophila* demonstrate precise sensory discrimination of cholesterol at the point of ingestion. Mammalian systems, in contrast, buffer fluctuations in dietary sterol availability through endogenous synthesis, centralized metabolic control, and intracellular sterol-sensing mechanisms. A tier-by-tier comparison of cholesterol homeostasis in mammals and insect taxa is summarized (Table 2).

Cholesterol occupies a central position in mammalian physiology, serving as an indispensable structural component of cellular membranes [115,116,117,118] and as the precursor for steroid hormones [34,35,36], bile acids [31,119], vitamin D [120,121], and multiple oxysterol-mediated signaling pathways [122,123,124]. Because fluctuations in cholesterol levels can perturb membrane dynamics, metabolic homeostasis, and modulate immune–endocrine functions, mammalian systems have evolved stringent mechanisms to monitor and regulate sterol abundance internally across tissues [125,126,127]. Mammalian homeostasis relies on an integrated, predominantly post-ingestive regulatory network encompassing *de novo* synthesis, intestinal absorption, intracellular trafficking, lipoprotein-mediated transport, storage, and efflux—each layer governed by transcriptional and post-transcriptional checkpoints [97,128,129,130,131,132]. Systemic cholesterol levels are sustained through two major inputs: endogenous synthesis via the mevalonate pathway and exogenous dietary intake [10]. These pathways converge on intracellular sterol-sensing regulatory nodes such as Sterol Regulatory Element-Binding Protein 2 (SREBP2), liver X receptors (LXRs), and the Low-Density Lipoprotein Receptor (LDLR)–lipoprotein axis, which collectively ensure adequate supply while preventing cytotoxic sterol accumulation, thereby buffering dietary variability [36,37]. This well-defined mammalian framework provides essential context for understanding sterol-auxotrophic insects [133], which lack endogenous cholesterol biosynthesis, have evolved fundamentally different strategies that externalize sterol regulation to the level of sensory detection and feeding behavior.

In mammals, intracellular cholesterol production through the mevalonate pathway constitutes a central buffering mechanism that stabilizes sterol availability independently of dietary intake. The rate-limiting step of this pathway is catalyzed by 3-hydroxy-3-methylglutaryl–CoA reductase (HMGCR) [134,135], whose expression and activity are tightly governed by SREBPs, with SREBP-2 acting as the principal transcriptional regulator of cholesterol biosynthesis [107,114]. SREBP-2 is synthesized as an endoplasmic reticulum–anchored precursor in complex with SREBP cleavage-activating protein (SCAP) and functions as an intracellular sterol sensor: under sterol-depleted conditions, the SCAP–SREBP complex translocates to the Golgi, where sequential cleavage by Site-1 protease (S1P) and Site-2 protease (S2P) releases the active N-terminal transcription factor [104,109]. Nuclear SREBP-2 subsequently induces coordinated expression of genes involved in cholesterol synthesis and uptake, including HMGCR and the LDLR, thereby restoring intracellular sterol levels [107,108,114]. Conversely, when cholesterol is abundant, sterol-bound SCAP recruits insulin-induced gene (INSIG) proteins to retain SREBP-2 in the ER, establishing a robust negative-feedback loop that suppresses further synthesis [110,111,113]. This transcriptional circuit is reinforced by post-translational control, whereby excess sterols accelerate HMGCR degradation via INSIG-dependent recruitment of the Gp78 and HRD1 E3 ubiquitin ligases through the ER-associated degradation (ERAD) pathway [136]. Together, these layered intracellular control mechanisms enable mammals to dynamically adjust cholesterol production according to cellular demand, effectively buffering fluctuations in dietary sterol availability.

Mammalian cells acquire extracellular cholesterol primarily through LDL particles, which are internalized via LDLR-mediated endocytosis [96,97,137]. This receptor-mediated uptake provides a tightly regulated, predominantly post-ingestive route for sterol acquisition that is decoupled from immediate dietary composition. After endosomal trafficking, cholesteryl esters are hydrolyzed by lysosomal acid lipase (LIPA) to release free cholesterol, which is further exported from lysosomes by the Niemann–Pick proteins NPC1 and NPC2—a conserved intracellular trafficking step that ensures efficient mobilization of LDL-derived sterols independent of sensory-level nutrient detection [138]. Excess intracellular cholesterol is rapidly re-esterified by the acyl-CoA: cholesterol acyltransferases ACAT1 and ACAT2 [also referred to as Sterol O-Acyltransferase 1/2 (SOAT1/2)], allowing its sequestration in lipid droplets and buffering transient sterol excess to prevent membrane toxicity [139].

Systemically, hepatocytes can also import cholesterol esters from high-density lipoprotein (HDL) through Scavenger Receptor class B member 1 (SR-BI)–mediated selective uptake, facilitating centralized hepatic control of whole-body sterol flux [140]. Within hepatocytes and macrophages, cholesteryl esters stored in lipid droplets can be mobilized by neutral lipases such as Adipose Triglyceride Lipase (ATGL) and Hormone-Sensitive Lipase (HSL), providing an internal reservoir that smooths fluctuations in sterol supply and demand [141].

To prevent toxic sterol accumulation, cells rely on ABC transporters for efflux. ABCA1 and ABCG1 mediate the transfer of cholesterol and phospholipids to nascent or mature HDL particles, constituting the core of macrophage-to-feces reverse cholesterol transport [142,143]. These genes are transcriptionally controlled by LXRs, which respond to oxysterol signaling to enhance cholesterol elimination and link intracellular sterol sensing to systemic disposal pathways [112]. In the liver and intestine, the ABCG5/ABCG8 heterodimer selectively excretes cholesterol—and plant sterols—into bile and the intestinal lumen, representing the principal pathway for whole-body sterol disposal [144]. LDLR abundance at the cell surface is further modulated by Proprotein convertase subtilisin/kexin type 9 (PCSK9)-mediated lysosomal degradation, a pathway that critically shapes plasma LDL-cholesterol levels and fine-tunes sterol uptake without reliance on behavioral avoidance or attraction to cholesterol-rich foods [145,146]. Together, LDLR-mediated uptake, intracellular trafficking via NPC1/NPC2, SR-BI–dependent exchange, and LXR-driven ABC transporter efflux form an integrated, internally regulated network that maintains cholesterol balance at cellular and systemic levels, thereby obviating the need for sensory-level cholesterol discrimination characteristic of sterol-auxotrophic insects.

## 4. Cholesterol Acquisition and Utilization Across Life Stages in *Drosophila*

**Figure 4:**
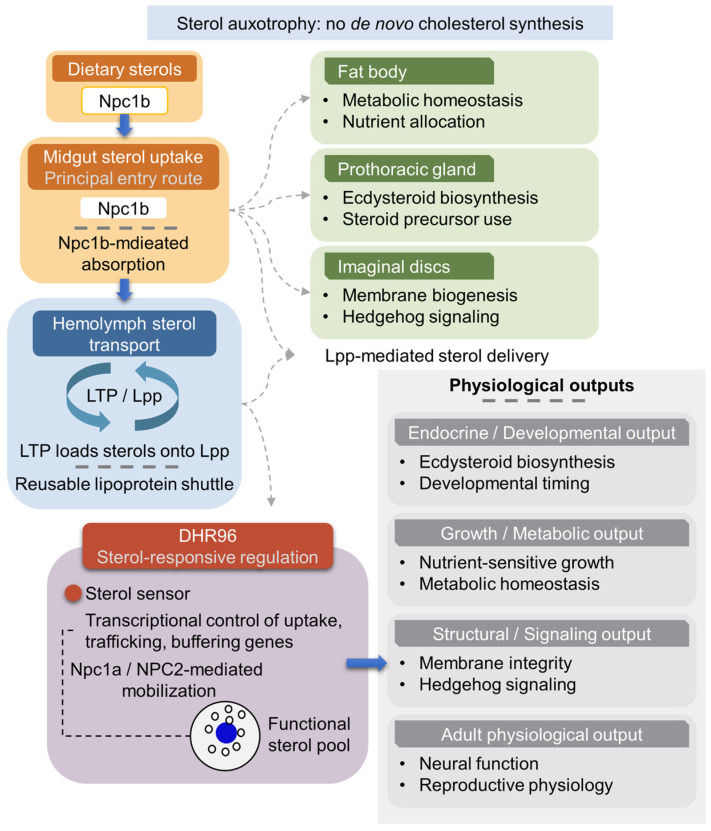
Conversion of dietary cholesterol into systemic endocrine and nutrient signals in *D. melanogaster*. Because *Drosophila* lacks *de novo* cholesterol biosynthesis, sterol homeostasis is organized around the acquisition, allocation, and functional utilization of dietary sterols. Dietary cholesterol is absorbed from the intestinal lumen through the midgut epithelium via Npc1b (Niemann–Pick type C1b)-mediated uptake, representing the principal entry route for sterols into the organism. Following uptake, cholesterol is loaded onto circulating lipophorin (Lpp) particles through the action of lipid transfer particle (LTP), which together function as a reusable shuttle to distribute sterols among peripheral tissues according to physiological demand rather than long-term storage. At the cellular level, sterols delivered to target tissues are mobilized from endo-lysosomal compartments by Npc1a (Niemann–Pick type C1a) and NPC2 (Niemann–Pick type C2) family proteins into a functional sterol pool that supports membrane integrity and signaling processes. In parallel, DHR96 (*Drosophila* hormone receptor 96) acts as a transcriptional sterol sensor, responding to intracellular sterol availability to adjust sterol uptake, transport, and buffering capacity. These allocation and sensing mechanisms collectively channel dietary cholesterol into distinct systemic outputs, including ecdysteroid-dependent developmental timing and endocrine control, nutrient-sensitive growth and metabolic homeostasis, and structural and signaling roles such as Hedgehog modification and neural function. Through this architecture, environmental sterol availability is directly coupled to organismal physiology in sterol-auxotrophic insects.

In striking contrast to mammals, which balance endogenous synthesis with dietary uptake, *D. melanogaster* is a sterol auxotroph and relies on sterols obtained from the external environment. The pathway linking dietary sterol uptake to systemic transport, intracellular mobilization, and endocrine/metabolic outputs in *Drosophila* is outlined (Figure 4). This fundamental constraint has driven the evolution of specialized mechanisms for sterol absorption, systemic transport, and intracellular trafficking, ensuring a continuous supply of precursors for membrane biogenesis and ecdysteroid production [147].

As a result, cholesterol regulation in insects is centered not on synthesis but on the efficient acquisition, allocation, and redistribution of dietary sterols across tissues with high metabolic or endocrine demand. Recent studies have begun to delineate the molecular architecture of these pathways—from gut-specific sterol uptake mechanisms to Lpp-based systemic transport and conserved intracellular regulators such as NPC1/2, ABC transporters, and the nuclear receptor DHR96—revealing both deep evolutionary conservation and insect-specific innovations in sterol homeostasis [41,102,103,105,106,148,149,150].

From a holistic scope, insect sterol metabolism is also relevant to poultry nutrition, as insect-based ingredients are potentially being evaluated as alternative feed resources [151,152]. Present data indicates that sterol composition varies substantially among insect species and is further influenced by rearing substrate; for instance, *Hermetia illucens* generally contains lower cholesterol and higher campesterol and β-sitosterol levels than several other edible insects, and its sterol profile can be modified by larval diet [153,154]. Additionally, current poultry studies, particularly in laying hens fed *H. illucens* meal, have not identified consistent detrimental effects on circulating cholesterol-related parameters and in some cases have reported reductions in serum cholesterol and triglycerides [151,152]. However, the precise mechanistic contribution of insect-derived sterols to poultry lipid metabolism remains insufficiently understood.

Here, we synthesize current knowledge on how *Drosophila* absorbs, traffics, and utilizes sterols across developmental stages, providing a framework for understanding the unique constraints and regulatory strategies that distinguish insect cholesterol metabolism from that of mammals. An integrated module-based summary of cholesterol handling across tissues, life stages, and physiological outputs is provided (Table 3).

Given sterol auxotrophy, sterol acquisition is thus a decisive determinant of larval growth, molting, and survival. Classical studies demonstrated that larvae deprived of exogenous sterols fail to progress beyond early developmental stages and ultimately die, a finding later reinforced by molecular analyses showing that dietary sterols provide the substrate for 7-dehydrocholesterol formation and the downstream ecdysone biosynthetic cascade [29,156,158]. Absorption occurs primarily in the midgut, where dietary cholesterol and phytosterols are internalized, processed, and allocated into tissue sterol pools required for growth, metamorphosis, and endocrine output. This dependence on environmental sterols establishes the foundation for the specialized uptake and transport mechanisms that distinguish insect sterol metabolism from mammalian systems [105].

In insects, systemic lipid trafficking is coordinated primarily by Lpp, a high-capacity circulating lipoprotein that functions as a reusable shuttle rather than being internalized and degraded like mammalian LDL. Lpp is composed primarily of apolipophorin-I/II and apolipophorin-III and operates analogously to mammalian HDL by repeatedly acquiring and delivering lipid cargo throughout the hemolymph [98]. After dietary uptake in the midgut, Lpp transports diacylglycerol, phospholipids, and sterols to peripheral tissues, including the fat body, imaginal discs, and the prothoracic gland, where sterols serve as essential precursors for ecdysteroid biosynthesis [29]. This process is facilitated by the lipid transfer particle (LTP), which selectively loads dietary cholesterol and phytosterols onto Lpp to enable their systemic distribution [99]. Cellular uptake of Lpp-derived lipids occurs through dedicated Lpp receptors (LpR1 and LpR2), ensuring efficient delivery of sterols to tissues with high metabolic and endocrine demands [159]. Together, Lpp and LTP form the core of the insect sterol transport machinery, supporting sterol delivery to peripheral tissues and enabling developmental progression despite the absence of endogenous synthesis.

The cellular mechanisms underlying sterol uptake and intracellular distribution in *Drosophila* are increasingly well-resolved, revealing a suite of conserved lipid-handling proteins adapted to an organism that lacks endogenous sterol biosynthesis. These pathways coordinate the acquisition of dietary cholesterol from Lpp particles, its mobilization from endosomal and lysosomal compartments, and its integration into endocrine and metabolic processes. *Drosophila* encodes two Niemann–Pick type C1 homologs with distinct physiological roles. Npc1b functions at the gut epithelium as a key determinant of dietary sterol absorption, whereas Npc1a acts broadly to mobilize sterols from late endosomes/lysosomes for tissue use, including ecdysteroidogenesis [102,103]. Loss of *Npc1a* leads to sterol accumulation and early lethality, while *Npc1b* mutants exhibit severe sterol malabsorption and developmental arrest [103].

The *Drosophila* genome contains eight *Npc2* paralogs (*Npc2a*–*Npc2h*), reflecting an expanded and functionally diversified sterol-binding system. Among these, *Npc2a* and *Npc2b* are partially redundant and support systemic sterol homeostasis and ecdysteroidogenesis. Several NPC2 proteins show compartment-specific expression, with some acting in lysosomal sterol export and others functioning extracellularly, suggesting functional specialization in sterol binding and transfer within the endo-lysosomal pathway [41]. Although less extensively characterized than their mammalian counterparts, several ABC transporters in *Drosophila*, particularly within the ABCG family, are thought to contribute to sterol efflux and membrane remodeling, potentially complementing NPC-mediated sterol mobilization, although their precise roles remain an active area of investigation [105].

The nuclear receptor DHR96 functions as an insect cholesterol sensor and serves as a functional analog of vertebrate LXR. DHR96 binds dietary sterols and orchestrates transcriptional responses that maintain sterol balance during both deficiency and excess. Loss of DHR96 renders flies hypersensitive to cholesterol deprivation and impairs their ability to tolerate high-cholesterol diets [105]. Genome-wide transcriptional profiling has identified Npc1b, Npc2a, and additional sterol transport and metabolism genes as direct transcriptional targets of DHR96. Recent data further suggest that DHR96 interacts with the SREBP pathway, integrating sterol sensing with broader lipid homeostasis [106].

**Figure 5:**
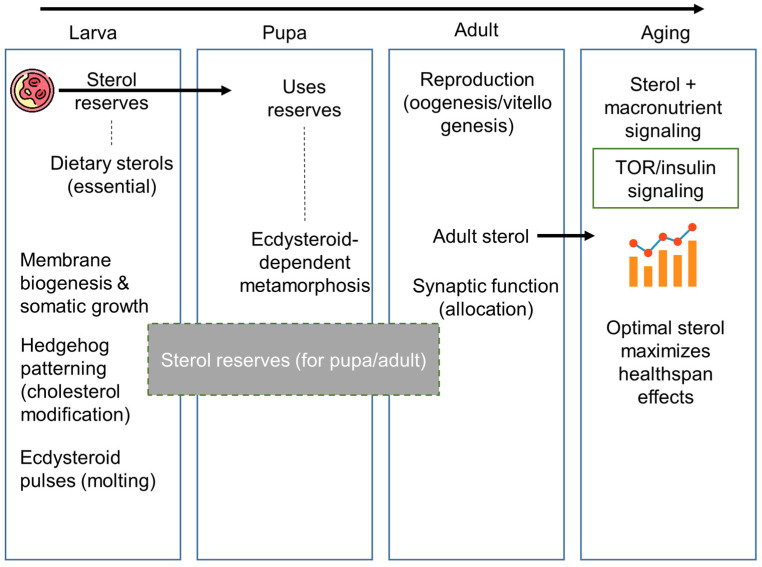
Stage-specific cholesterol dependence and life-history trade-offs in *D. melanogaster.* Cholesterol functions as a pleiotropic, stage-dependent resource across the *Drosophila* life cycle, with distinct physiological outcomes determined by developmental timing and nutritional context. During larval development, dietary sterols are essential to support membrane biogenesis and somatic growth, Hedgehog-dependent developmental patterning through cholesterol modification, and repeated ecdysteroid pulses that drive molting. In parallel, excess dietary sterols are accumulated as reserves that support subsequent developmental stages; limitation of sterol availability during larval growth leads to developmental arrest or lethality. The pupal stage represents a strict metabolic bottleneck, as feeding ceases and all endocrine signaling and metamorphic remodeling depend exclusively on sterol reserves acquired during larval life, rendering survival sensitive to reserve availability through ecdysteroid-dependent metamorphosis. In adults, cholesterol is continuously allocated among competing physiological demands, including ecdysteroid-dependent reproductive processes, neural membrane maintenance, and synaptic function, giving rise to allocation trade-offs rather than further growth. During aging, dietary cholesterol interacts with macronutrient balance and nutrient-sensing pathways, including TOR and insulin signaling, producing non-linear effects on healthspan that reflect trade-offs between somatic maintenance and reproduction.

Together, these conserved molecular systems highlight how *Drosophila* achieves efficient sterol uptake and intracellular handling despite complete dependence on environmental sterols, revealing both conceptual parallels with mammalian cholesterol trafficking and a fundamental shift from synthesis-buffered control to acquisition- and allocation-driven regulation imposed by sterol auxotrophy. Stage-specific cholesterol dependence and life-history trade-offs are summarized (Figure 5).

### 4.1. Larval Stage

The larval stage represents the fastest period of growth in *Drosophila*, characterized by intense feeding and the accumulation of macromolecular reserves that support metamorphosis [160]. In sterol-auxotrophic insects, this growth phase places exceptional demands on dietary cholesterol, which fulfills dual structural and endocrine functions. Sterols are required for membrane biogenesis and cellular proliferation, and they serve as the sole precursors for ecdysteroids, the steroid hormones that orchestrate larval molting and the larva-to-pupa transition [29,156]. Recent studies further show that cholesterol availability acts as a nutritional signal that couples tissue growth to developmental timing through steroidogenic output and TOR-dependent nutrient signaling, positioning cholesterol as both a metabolic substrate and a regulatory cue during larval maturation [48].

Beyond its role in endocrine signaling, cholesterol directly modulates larval tissue patterning through its involvement in morphogen signaling pathways. Among these, Hedgehog (Hh) provides a prominent example of sterol-dependent regulation [40]. In *Drosophila*, Hh undergoes an unusual post-translational modification in which its C-terminal domain is autocatalytically cleaved and covalently linked to cholesterol, while the N-terminus is palmitoylated [161]. This dual lipidation anchors Hh to membranes and governs its spatial distribution, enabling proper gradient formation across imaginal discs and ensuring correct anterior–posterior patterning and growth [162,163]. Thus, cholesterol contributes not only to hormone production but also to the spatial control of developmental signaling during larval growth.

A central function of cholesterol during larval development is its role as the indispensable precursor for ecdysone biosynthesis. Developmental progression in *Drosophila* is governed by temporally regulated pulses of the steroid hormone 20-hydroxyecdysone (20E), which coordinate larval molts and commit the organism to metamorphosis. Because insects cannot synthesize sterols *de novo*, all ecdysteroids are ultimately derived from dietary cholesterol or plant sterol precursors [156]. Accordingly, the magnitude and timing of ecdysteroid pulses are tightly constrained by sterol supply during larval feeding.

Ecdysteroid biosynthesis occurs in the prothoracic gland through a conserved multi-step steroidogenic cascade involving enzymes encoded by the Halloween genes, including cytochrome P450 monooxygenases and associated dehydrogenases [164,165,166,167,168,169]. Rather than detailing each enzymatic step, it is critical to note that this entire pathway is substrate-limited by cholesterol availability. The first committed step is the conversion of cholesterol to 7-dehydrocholesterol by the Rieske oxygenase Neverland [155], followed by a series of poorly resolved transformations (“the Black Box”) and subsequent hydroxylations that yield ecdysone and finally 20E. Efficient delivery of cholesterol to this pathway depends on intracellular trafficking proteins such as Npc1a and accessory factors including Noppera-bo, which collectively channel dietary sterols toward hormone biosynthesis [102,170].

Limiting sterol intake reduces ecdysteroid output, delays developmental timing, and leads to growth arrest and larval lethality [171]. These outcomes underscore a defining feature of insect physiology: developmental progression is not buffered by endogenous synthesis but is instead directly coupled to dietary sterol availability.

### 4.2. Pupal Stage

The pupal stage represents a phase of profound morphological reorganization, during which larval tissues undergo programmed cell death and are replaced by adult structures derived from imaginal discs and histoblasts [172]. This transformation is orchestrated by precisely timed pulses of 20E that initiate pupariation and coordinate tissue remodeling [173,174]. Crucially, feeding ceases at the onset of pupariation, rendering pupal development entirely dependent on sterol reserves accumulated during larval life.

Because all ecdysteroids required for metamorphosis derive from these pre-existing sterol stores, the pupal stage represents a physiological bottleneck in which prior cholesterol acquisition directly determines developmental success. In this way, larval nutrition is translated into pupal endocrine competence, linking early dietary conditions to irreversible developmental outcomes [29,173].

### 4.3. Adult Stage

Following eclosion, the role of cholesterol shifts from supporting developmental transitions to maintaining adult physiological homeostasis, reproductive capacity, and neural function. Cholesterol remains an essential structural component of cellular membranes throughout adult life, contributing to membrane fluidity and the proper function of membrane-associated signaling complexes. As in earlier stages, systemic sterol distribution in adults relies on Lpp particles, enabling sterol delivery to metabolically active tissues such as the fat body, intestine, germline, and nervous system [99].

Steroid hormone signaling continues to play central roles in adulthood. The active ecdysteroid 20E, acting through the Ecdysone receptor/Ultraspiracle nuclear receptor complex, regulates reproduction, feeding behavior, stress responses, and lifespan [175]. In females, 20E promotes vitellogenin expression and uptake into developing oocytes. The perturbations in sterol availability or ecdysteroid signaling impair egg maturation and fertility [176]. Thus, dietary sterols acquired during development and adulthood collectively determine reproductive output long after metamorphosis.

Cholesterol is also indispensable for adult neural function. Disruption of sterol trafficking pathways, including mutations in ABCG-family transporters, leads to defects in synaptic performance and impairments in olfactory learning and memory [157,177]. These findings highlight the sensitivity of the adult nervous system to sterol imbalance and demonstrate that cholesterol-dependent processes remain essential for higher-order behavior beyond developmental patterning.

### 4.4. Cholesterol, Nutrient Signaling, and Lifespan

Dietary composition is a major determinant of lifespan, and cholesterol plays a uniquely influential role in shaping life-history trajectories in *Drosophila*. As an essential but non-synthesizable micronutrient, cholesterol supports membrane homeostasis, endocrine signaling, and reproductive investment, thereby influencing both longevity and fecundity. Studies using holidic diets reveal that cholesterol exerts non-linear, context-dependent effects on lifespan, characterized by a hump-shaped dose–response relationship [49,178]. Severe sterol limitation shortens lifespan and compromises reproduction, whereas moderate cholesterol supplementation restores survival and fertility. At higher concentrations, however, excess sterol intake can impair metabolic balance and diminish lifespan benefits.

Importantly, cholesterol’s effects on lifespan are strongly modulated by interactions with macronutrients. High protein-to-carbohydrate diets amplify sensitivity to cholesterol availability, suggesting that sterol requirements scale with reproductive effort and anabolic signaling. Thus, cholesterol acts not in isolation but as an integral component of a broader nutritional signaling network [49].

Mechanistically, cholesterol has emerged as a regulator of growth and lifespan through the Npc1–TOR–insulin signaling axis. Dietary sterols activate Npc1-dependent trafficking in the fat body and blood–brain barrier, stimulating TOR signaling and promoting insulin-like peptide secretion from insulin-producing neurons [48]. While moderate activation of this pathway supports growth and homeostasis, excessive Insulin/insulin-like growth factor signaling/TOR signaling accelerates aging, providing a mechanistic explanation for the non-linear effects of cholesterol on lifespan. In this framework, cholesterol functions simultaneously as an essential biochemical substrate and a regulatory nutrient signal that shapes aging through endocrine–metabolic integration.

## 5. Conclusions

Together, mammalian and insect studies reveal two contrasting yet complementary strategies for cholesterol detection: receptor modulation in mammals versus dedicated gustatory receptors in insects. These differences likely reflect divergent ecological pressures, including sterol auxotrophy in insects and endogenous cholesterol synthesis in mammals. Key open questions remain. In mammals, it is unclear whether cholesterol-responsive taste modulation contributes to conscious perception or primarily shapes feeding behavior indirectly. In insects, whether cholesterol-sensing IR complexes are conserved across species or tuned to specific sterol derivatives remains unexplored. It remains unknown whether cholesterol sensing differs across developmental stages. Given the essential role of cholesterol during early development, larvae must acquire sufficient amounts to support growth, which would predict an associated attractive behavioral valence. Thus, stage-specific modulation of cholesterol preference is plausible, yet currently unresolved. This possibility is supported by precedent: *Drosophila* larvae are attracted to ribose, whereas adults exhibit aversion to the same compound, indicating that chemosensory valence can be developmentally reprogrammed [179,180]. More broadly, these findings prompt a reassessment of how non-canonical nutrients are incorporated into taste coding strategies and highlight cholesterol gustation as a model for understanding how metabolic necessity intersects with sensory evolution.

From a translational perspective, the expanded use of insect-derived ingredients in animal nutrition also points out important practical limitations. Although insect meals are promising sustainable feed alternatives, their nutritional value is not uniformly positive across all formulations or inclusion levels. In poultry, higher dietary inclusion of black soldier fly meal has been associated with poorer feed conversion and adverse changes in intestinal morphology, including reduced villus height and villus height-to-crypt depth ratio [181,182]. Digestibility can also vary with the degree of defatting and processing [183]. These constraints are often attributed, at least in part, to the chitin fraction, which may reduce nutrient availability, particularly at high inclusion levels [183,184]. Accordingly, future work should define species-specific tolerance thresholds and determine how insect species, processing methods, and dietary context shape digestibility, gut health, and metabolic outcomes in target animals.

## Figures and Tables

**Table 1:** Cholesterol sensation in mammals and insects.

Model Organism	Cholesterol Acquisition	Sensory Regulation	Peripheral Requirement	Coding Logic	Behavioral Output	Key References
Mouse (Mammal)	Endogenous synthesis	Modulatory cue	TAS2R14 ^a^ (bitter) cholesterol interaction	Physiological modulation	Context-dependent preference	[30,87,88,92,93]
*Drosophila melanogaster* (Insect)	Dietary dependence	Dedicated gustatory stimulus	IR ^b^ complexes in GRNs ^c^	Labeled-line encoding	Concentration-dependent attraction or avoidance	[29,39,74,94]

^a^ taste receptor type 2 member 14. ^b^ ionotropic receptor. ^c^ gustatory receptor neurons.

**Table 2:** Evolutionary divergence in cholesterol homeostasis.

Feature	Mammals	*Drosophila melanogaster*	Key References
Sterol sourcing	Dual source: synthesis + diet ^a^	Dietary obligate (auxotroph)	[38,39,92]
Systemic transport	LDL–LDLR pathway ^b^	Lipophorin-mediated transport ^c^	[95,96,97,98,99]
Intracellular routing	NPC1/NPC2 mobilization ^d^	Npc1a/Npc1b dependent roles ^d^	[100,101,102,103]
Transcriptional sterol sensing	SREBP–SCAP–INSIG; LXR ^e^	DHR96-dependent sterol response ^f^	[104,105,106,107,108,109,110,111,112,113,114]

^a^ Mevalonate pathway–based *de novo* cholesterol synthesis. ^b^ LDL internalization via LDL receptor–mediated endocytosis. ^c^ Lipophorin enables sterol exchange without lysosomal degradation. ^d^ Conserved NPC1/2-mediated intracellular sterol trafficking. ^e^ Liver X receptor (LXR) senses intracellular sterols/oxysterols. ^f^ DHR96: *Drosophila* nuclear receptor regulating dietary sterol response.

**Table 3:** Cholesterol functional roles in *Drosophila*.

Process	Feature	Primary Tissue(s)	Life Stage	Functional Output	Key References
Gustation	IR complexes ^a^	Labellar GRNs ^b^	Larva, adult	Sterol taste coding	[74]
Intestinal uptake	Npc1b ^c^	Midgut enterocytes	Larva, adult	Entry of dietary sterols from the gut epithelium	[103]
Systemic transport	Lipophorin ^d^	Hemolymph	All developmental stages	Circulation of sterol	[98,99]
Intracellular mobilization	Npc1a/Npc2 ^e^	Fat body, CNS	All developmental stages	Lysosomal sterol mobilization	[41,102]
transcriptional control	DHR96 ^f^	Gut, fat body	Larva, adult	Sterol-responsive gene regulation	[105,106]
Morphogen signaling	Hedgehog ^g^	Imaginal discs	Larva	Cholesterol-modified Hh signaling	[40]
Ecdysteroid synthesis	*neverland*; Halloween genes ^h^	Prothoracic gland	Larva, pupa	Steroid hormone production	[155,156]
Neural function and lifespan regulation	Npc1a; IIS–TOR ^i^	CNS	Adult	Sterol-dependent neural physiology	[41,48,49,157]

^a^ ionotropic receptor family. ^b^ gustatory receptor neurons. ^c^ Npc1b: intestinal sterol transporter. ^d^ Lipophorin (Lpp): major hemolymph lipid carrier. ^e^ Npc1a/Npc2: intracellular sterol trafficking proteins. ^f^ DHR96: nuclear receptor regulating dietary sterol response. ^g^ Hedgehog (Hh): cholesterol-modified morphogen. ^h^ *neverland* and Halloween genes: enzymes in ecdysteroid biosynthesis. ^i^ IIS–TOR: insulin/target of rapamycin signaling axis.

## Data Availability

No new data were created or analyzed in this study. Data sharing is not applicable to this article.
